# Development of an *in vitro* Microtuberization and Temporary Immersion Bioreactor System to Evaluate Heat Stress Tolerance in Potatoes (*Solanum tuberosum* L.)

**DOI:** 10.3389/fpls.2021.700328

**Published:** 2021-08-11

**Authors:** Sanjeev Gautam, Nora Solis-Gracia, Megan K. Teale, Kranthi Mandadi, Jorge A. da Silva, M. Isabel Vales

**Affiliations:** ^1^Department of Horticultural Sciences, Texas A&M University, College Station, TX, United States; ^2^Texas A&M AgriLife Research & Extension Center at Weslaco, Weslaco, TX, United States; ^3^Department of Plant Pathology and Microbiology, Texas A&M University, College Station, TX, United States; ^4^Department of Soil and Crop Science, Texas A&M University, College Station, TX, United States

**Keywords:** high temperature, abiotic stress, climate change, heat tolerance, tuberization media

## Abstract

High temperature (heat) stress reduces tuber yield and quality of potatoes. Screening potatoes for heat tolerance is increasingly important, considering the climate change scenario and expansion of potatoes to countries where heat stress is an issue. *In vitro* screening for tolerance to abiotic stresses offers several advantages, including quick evaluation of numerous genotypes (clones) in reduced space, controlled environmental conditions (temperature and photoperiod), and free from confounding variables inherent to greenhouse and field conditions. In this study, we explored the feasibility of using a temporary immersion bioreactor system for heat tolerance screening of potatoes. We determined the best hormone-free microtuberizing media for this system (MSG with 8% sucrose) to enhance microtuber number and size. Comparisons of microtubers produced at 30°C as heat treatment, with 16°C as normal condition, allowed to identify heat tolerant and susceptible potato clones. The use of bioreactors allowed distinguishing well-formed (non-deformed) from deformed microtubers. Heat stress increased the total biomass of plant tissues in all the clones. However, the effect of heat stress on microtuber number and weight varied among the clones. Incubation at 30°C decreased the weight and number of non-deformed microtubers in all the clones except for Reveille Russet in which the weight of non-deformed microtubers was significantly increased and the count of non-deformed microtubers was not affected. The potato variety Reveille Russet, which was selected under high-temperature field conditions in Texas, had many non-deformed microtubers per explant and the highest microtuber weight among four clones evaluated under heat stress. We described a faster and reliable *in vitro* microtuberization system for abiotic stress tolerance screening, identified Reveille Russet as a promising heat-tolerant potato variety, and confirmed Russet Burbank and Atlantic as susceptible heat-tolerant checks.

## Introduction

Due to global climate change, extreme environmental conditions such as drought and heat have significantly damaged agricultural production and will continue to affect food production worldwide. In potato (*Solanum tuberosum L*.), heat stress is a significant limitation for production (Lehretz et al., 2019). It has been reported that if temperatures continue to rise in the future, substantial tuber reductions will occur in several potato-producing regions (Hijmans, 2003). Potato is a cool-season crop with optimum temperature for its growth at 20°C (Ahn et al., 2003). Dry matter accumulation is maximum within the range of 14–22°C. According to Mohabir and John (1988), the optimum temperature for starch formation in tubers is 21.5°C. Tuberization is reduced at night temperatures above 18°C and could be absent beyond 25°C (Bushnell, 1927) even though potato plants can tolerate day temperatures of about 32°C without significant loss in total biomass production (Minhas et al., 2006).

Physiological deformations of tubers, such as elongated tubers, bottlenecks, second-growth (chain tuberization), and gemmations, may also occur in potatoes grown in heat-stressed conditions (Rykaczewska, 2015). Overall, the negative consequences of heat stress in potatoes are often realized by reducing the yield of marketable tubers. In one of the studies to assess the effect of heat, a 56% reduction in marketable tuber ratio was observed (Kim et al., 2017). Not only is yield affected by heat stress, but the external and internal tuber quality is also affected. High soil temperature during tuber initiation, bulking, and maturation of potatoes alters postharvest carbohydrate metabolism to weaken genotypic resistance to cold-induced sweetening. It accelerates the loss of processing quality, even in cold sweetening resistant clones (Zommick et al., 2014). The number one variety in the US and Canada, Russet Burbank, performs poorly under high temperatures, which can be observed from the reduced production acreage in Ontario during the hot summer of 2016 (Tang et al., 2018). We can expect similar scenarios accompanied by the economic loss happening more frequently in the future if we experience higher temperatures.

The effects of heat stress vary according to genotype, occurrence, duration, and intensity of high temperature. Different potato cultivars differ in their responses to heat stress (Levy, 1986; Chandra et al., 2000; Ahn et al., 2003; Figueiredo et al., 2016). The development of varieties with tolerance is an effective way to mitigate the detrimental effect of heat stress, whether it is transient or long-term (Bonnel, 2008; Demirel et al., 2017; Busse et al., 2018). To develop heat-tolerant potato varieties, we need to know the level of heat tolerance in potato genotypes and other relevant traits so we can select the best parents for the breeding program. Screening potatoes under field conditions involves various fluctuating environmental factors apart from temperature, leading to variable and inconclusive results about the genotypes regarding their initial screening. Furthermore, screening of many genotypes would require more land and other resources. By tissue culture (*in vitro*), the effect of high temperature could be isolated from other environmental factors (Khan et al., 2015). Thus, *in vitro* screening for heat tolerance would have several advantages, such as screening many clones in a limited space and relatively quickly, which would save time and space (Nowak and Colborne, 1989). *In vitro* screening for heat tolerance would greatly benefit potato breeding by narrowing down the genotypes that have to be advanced and verified by follow-up studies in greenhouses and field trials. The *in vitro* screening mainly aims for the genotype's ability to microtuberize in high temperatures. The rationale is that the explants surviving or performing well in stress conditions provided *in vitro* can perform well under field conditions. High temperature *in vitro* screening has been achieved for potato clones and True Potato Seed (Khan et al., 2015; Guedes et al., 2019). The possibility of identifying heat-tolerant potato genotypes through *in vitro* screening has also been explored by other researchers (Gopal et al., 1998; Khan et al., 2015; Pantelić et al., 2018; Guedes et al., 2019). Several microtuberization protocols have been used with solid media or liquid media, with or without hormones, preconditioning with some chemicals or not. Most protocols fail to produce micro tubers that are larger in size (>0.5 g) (Vreugdenhil et al., 2007). *In vitro* microtuberization with liquid media has produced larger microtubers (Leclerc et al., 1994; Jimenez et al., 1999). However, the microtubers with liquid culture tend to be soft and develop open lenticels. Hyperhydricity results from poor exchange of gases when explants are continuously immersed in liquid medium (Etienne and Berthouly, 2002). Temporary immersion bioreactors have been used to efficiently produce potato microtubers (Kämäräinen-Karppinen et al., 2010; Tapia et al., 2018). A temporary immersion bioreactor system for microtuberization produced more and larger microtubers than in solid media (Jimenez et al., 1999) and did not experience the hyperhydricity observed in permanent liquid culture (Etienne and Berthouly, 2002). However, because different genotypes and cultivars respond uniquely to heat stress and various *in vitro* propagation media, the most suitable conditions for germplasm screening have to be empirically determined for other cultivars. This study was conducted to identify the best microtuberization media for potatoes grown *in vitro* and subsequently develop an *in vitro* microtuberization system to screen potato cultivars for heat tolerance using temporary immersion bioreactors placed inside plant growth chambers.

## Materials and Methods

### Plant Materials

The Texas A&M Potato Breeding Program provided disease-free tissue cultured plantlets. Five potato clones representing different market classes were used to identify of best tuberization media. The potato clones tested included: Russet Burbank (processing russet), Russet Norkotah (fresh russet), Atlantic (chipper), COTX05082-2P/P (fresh, purple skin, purple flesh), and COTX09022-3RuRE/Y (fresh, russet skin with yellow flesh). For the high-temperature screening experiments using bioreactors (Bioreactor experiments), four selected clones were used: Russet Burbank, Atlantic, Reveille Russet, and NDTX081648CB-13W. Russet Burbank is the number one russet processing variety in the USA. However, under high-temperature field conditions, it produces low total yield with low tuber number and low tuber weight. External defects such as knobs, growth cracks, and hollow hearts are prevalent under high-temperature conditions and result in low marketable yield. Atlantic is the most popular chipping variety in the USA, it produces relatively good yield under heat stress conditions, but the internal quality is seriously affected since heat stress causes internal heat necrosis of the tuber flesh, making the tubers unmarketable for making potato chips. Reveille Russet is a fresh market potato variety released by the Texas A&M Potato Breeding Program in 2015, selected under high-temperature field conditions in Texas. It has a good tuber appearance, high yield, and low internal and external defects, which results in high marketable yield under high-temperature conditions. Reveille Russet is quickly gaining acceptance in Texas, other parts of the USA, and Canada. NDTX081648CB-13W is an experimental advanced chipper clone from the Texas A&M Potato Breeding Program, also selected under high-temperature field conditions in Texas. Incidentally, NDTX081648CB-13W plants survived several days at >38°C in a greenhouse in College Station, Texas, and thus was considered a potential tolerant variety to high-temperature conditions.

### Establishment of *in vitro* Microtuberization System for Screening Heat Tolerance in Potatoes

#### Media Selection for Microtuberization

Five different *in vitro* media compositions were explored to identify the best microtuberizing media. The control medium was 4.4 g L^−1^ Murashige & Skoog with Gamborg's vitamins (MSG) with 30 g L^−1^ sucrose, and 8 g L^−1^ agar. The four remaining media were MSG supplemented with 80 g L^−1^ (or 8%) sucrose; MSG, with 8% sucrose, and 500 mg L^−1^ chlorocholine chloride (CCC); MSG with 8% sucrose, and 10 mg L^−1^ 6-benzylaminopurine (BAP); and MSG, with 8% sucrose, 500 mg L^−1^ CCC, and 10 mg L^−1^ BAP. The pH of each media was adjusted to 5.7, and all media were made using nanopure water and sterilized by autoclaving at 121°C for 20 min. The CCC was added to the media via filter sterilization to maintain its effectiveness.

The cultures were initiated from a 1 cm single node section and placed onto 10 ml of culture media held within a Pyrex No. 9820 (PYREX™) tube. Thirty nodes were cultured per condition, per treatment, and per variety (CTV). After rooting occurred, 20 healthy plantlets were selected from the 30 nodes initially cultured per CTV and grouped into repetitions based on similar height. The original 30 were grown to ensure an abundance of healthy material that would be roughly the same height when 20 were selected for each replication. Culture tubes were stored in lab conditions at 24–26°C under 32-watt Alto II Philips fluorescent lights on a 16:8 h light: dark photoperiod for 12 weeks. After the twelfth week, the total number of microtubers was counted, and the final microtuber diameters and weights were measured using a Vernier caliper and a calibrated scale.

#### Characterization of Heat-Sensitivity/Tolerance

Based on results from the media experiment, bioreactor experiments were planned. In order to reliably evaluate heat tolerance of tuberization, we performed two *in vitro* microtuberization assays using potato cultures growing in temporary immersion bioreactors (SETIS™, VERVITbvba, Zelzate, Belgium) under a controlled environment. There were two stages in each set of the experiments. The first stage was the vegetative growth and the second one was the tuberization stage.

The bioreactors were assembled according to the manufacturer specifications. Single node cuttings were excised from potato plantlets growing in MSG medium in tissue culture, and they were placed in the upper bioreactor vessel. In the first experiment, 25 cuttings were placed per bioreactor. Since culturing 25 explants found to be overcrowded, only 5–7 nodes were placed in the second experiment to keep the explants separated among themselves and avoid overcrowding. The explants were placed in 250 ml MSG (without sucrose) containing 2.5% gelrite as a solid base in the upper vessel of the bioreactor. The lower replaceable vessel of the bioreactor contained 750 mL of liquid medium. Bioreactor units were connected through SETIS™ 0.22 μm air filters to a tubing system, which was connected to an air compressor (Paasche, model D500SR, Chicago, IL), operating for 15 min every 4 h at an air pressure of 0.2 BAR, to conduct the temporary explant immersions in the upper culture vessels.

During the first 4 weeks stage, the plant tissues were exposed intermittently to growth liquid MSG media containing 30 g L^−1^ sucrose, under 16:8 (light: dark) photoperiod and at a constant 25 °C temperature to promote plant growth; during the second 8 weeks stage, the media vessels were replaced with new ones containing MSG with 80 g L^−1^ sucrose and the photoperiod was changed to 12:12 (light: dark) to promote tuberization. To explore the effect of the temperature in the tuberization, half of the bioreactors were exposed to a constant 16°C (optimum condition for tuber formation) and half of them to 30°C (heat stress) in Percival growth chambers model-36L5 (Percival Scientific, Inc., Perry, IA) under fluorescent lamps with a PPFD of 109 μmol m^−2^ sec ^−1^.

### Experimental Design and Analysis

The experiments were set up as factorial completely randomized designs. The media experiment included factor A: five different media and factor B: five different genotypes.

The bioreactor experiments were also set up as factorial completely randomized design with factor A: temperature with two levels (16 and 30°C). The four clones mentioned above were used as factor B. Three bioreactors were used for each clone per temperature. Therefore, a total of 2^*^4^*^3=24 bioreactors were used in each experiment. Half of the bioreactors were kept under 16°C (optimum condition for potato tuberization), and half of the bioreactors were moved to 30°C (heat stress) in Percival growth chambers.

Total biomass, non-tuber biomass, microtuber number, and microtuber weight were the primary observations recorded at the end of the experiment. However, during the harvesting of microtubers for the first bioreactor trial, some microtubers under 30°*C* were found misshaped and sprouted. This was considered a valuable trait, and in the second bioreactor trial, microtubers were harvested in different classes of deformed tubers and non-deformed tubers. The microtubers were separated into different classes based on appearance and size. The microtubers and the other parts were separated and weighed separately. They were oven-dried at 80°C until constant dry weights were achieved. The data were recorded and organized in a MS Excel spreadsheet. Data analysis was done using R-Version 4.0.3 (R Core Team, 2020). The packages used were agricolae (Mendiburu and Yaseen, 2020) and data.table (Dowle and Srinivasan, 2020). The graphs were prepared using the package ggplot2 (Wickham, 2016) and ggpubr (Kassambara, 2020). Two-way ANOVA was carried out for traits recorded at 0.05 level of significance, and means were separated using least significant difference (LSD). The traits evaluated are expressed on a per explant basis to make it easier to compare the results from the two sets of experiments since the first bioreactor trial had more (25) explants, as compared with the second bioreactor trial which had 5–7 explants per bioreactor. The reason for this decreased explant numbers in bioreactors was to avoid overcrowding. Typically, plants grow vigorously in bioreactors, and too many explants can overcrowd and make it difficult to separate them for data collection during the experiment.

## Results

### Media Experiment

The main objective of the experiment was to choose the best microtuberizing media for potatoes. Analysis of variance (Table 1) showed that there were significant differences (*p* ≤ 0.01) between media, clones, and their interaction for the characters on microtubers. Since the mean squares due to interactions were much lower than those due to media and clones, means separation for media was carried out. The number of microtubers produced per explant varied among the media and the clones tested. Overall, media with MSG plus 8% sucrose and without hormones gave the highest number of microtubers per explant, highest microtuber weight, and highest diameter per explant (Table 2) and thus, this media was used for subsequent experiments using bioreactors.

**Table 1:** Analysis of variance for *in vitro* tuberization of five selected potato clones^a^ in various microtuberization media.

**Character per explant**	**Mean squares**			
	**Media^**b**^**	**Clone**	Media*Clone	**Error**
Microtuber number (no.)	1.5546^**^	2.0666^**^	0.7141^**^	0.0583
Microtuber weight (g)	0.12781^**^	0.04882^**^	0.02138^**^	0.00745
Microtuber diameter (mm)	57.36^**^	36.20^**^	11.01^**^	3.33
df	4	4	16	72

a *Russet Burbank, Russet Norkotah, Atlantic, COTX05082-2P/P, and COTX09022-3RuRE/Y.*

b *MS with 8% sucrose, MS with 10 mg L^−1^ BAP and 8% sucrose, MS with 500 mg L^−1^ CCC and 8% sucrose.*

*MS with 10 mg L^−1^ BAP, 500 mg L^−1^ CCC and 8% sucrose, regular MS media with 3% sucrose.*

** *Significant at p ≤ 0.01*.

**Table 2:** Means over clones^1^ for various microtuber characters in different media.

**Character per explant**	**Media** ^**2**^					
	**S**	**BS**	**CS**	**BCS**	**MSG**	**LSD^**3**^**
Microtuber number (no.)	0.74^a^	0.22^c^	0.53^b^	0.02^d^	0.31^c^	0.152^**^
Microtuber weight (g)	0.209^a^	0.041^c, d^	0.124^b^	0.003^d^	0.074^b, c^	0.054^**^
Microtuber diameter (mm)	4.64^a^	1.61^c^	3.32^b^	0.18^d^	2.37^b, c^	1.151^**^

1 *Russet Burbank, Russet Norkotah, Atlantic, COTX05082-2P/P, and COTX09022-3RuRE/Y.*

2 *S: MS with 8% sucrose; BS: MS with 10 mg L^−1^ BAP and 8% sucrose; CS: MS with 500 mg L^−1^ CCC and 8% sucrose; BCS: MS with 10 mg L^−1^ BAP, 500 mg L^−1^ CCC and 8% sucrose; MSG: Regular MS media with 3% sucrose.*

3 *LSD at 0.05. Values followed by the same letter in a row were not statistically different.*

** *Significant at p ≤ 0.01*.

### Bioreactor Experiments

#### Total Biomass

First bioreactor trial: There was significant interaction between temperature and clones, indicating that differential performance of clones at optimum vs. high-temperature-stress conditions. Overall, high-temperature stress increased the total biomass production of the clones; also total biomass production varied significantly between clones (Table 3).

**Table 3:** Effect of temperature on growth and microtuber yield (per explant) of potato clones in temporary immersion bioreactor bioreactors (first bioreactor trial).

**Temp. (^**°**^C)**	**Clone**	**Total Dry Weight^**1**^ (g)**	**Non-tuber dry weight (g)**	**Total number of microtubers (no.)**	**Dry weight of all microtubers (g)**
16	Atlantic	0.825^b^	0.574^c^	3.627^b^	0.250^b^
30	Atlantic	0.842^b^	0.836^a, b^	0.093^e^	0.006^e^
16	NDTX081648CB-13W	0.919^a, b^	0.644^b, c^	5.067^a^	0.275^a, b^
30	NDTX081648CB-13W	1.112^a^	1.051^a^	0.720^e^	0.061^d, e^
16	Reveille Russet	0.317^c^	0.177^d^	2.640^c^	0.141^c, d^
30	Reveille Russet	0.478^c^	0.102^d^	1.620^d^	0.376^a^
16	Russet Burbank	0.393^c^	0.262^d^	5.600^a^	0.131^c, d^
30	Russet Burbank	0.805^b^	0.622^b, c^	1.800^d^	0.183^b, c^
	LSD ≤ 0.05	0.241	0.226	0.718	0.109
	Temperature	^**^	^*^	^**^	ns
	Clone	^**^	^**^	^**^	^*^
	Temperature*Clone	^*^	^*^	^**^	^**^

^1^ *Values followed by the same letter in a column are not statistically different as analyzed by LSD test at alpha level of 0.05. LSD values represent the interaction term for temperature and clone.*

*, ** *Indicate significant at p ≤ 0.05, 0.01, respectively, and ns indicates non-significant at p ≤ 0.05*.

**Figure 1:**
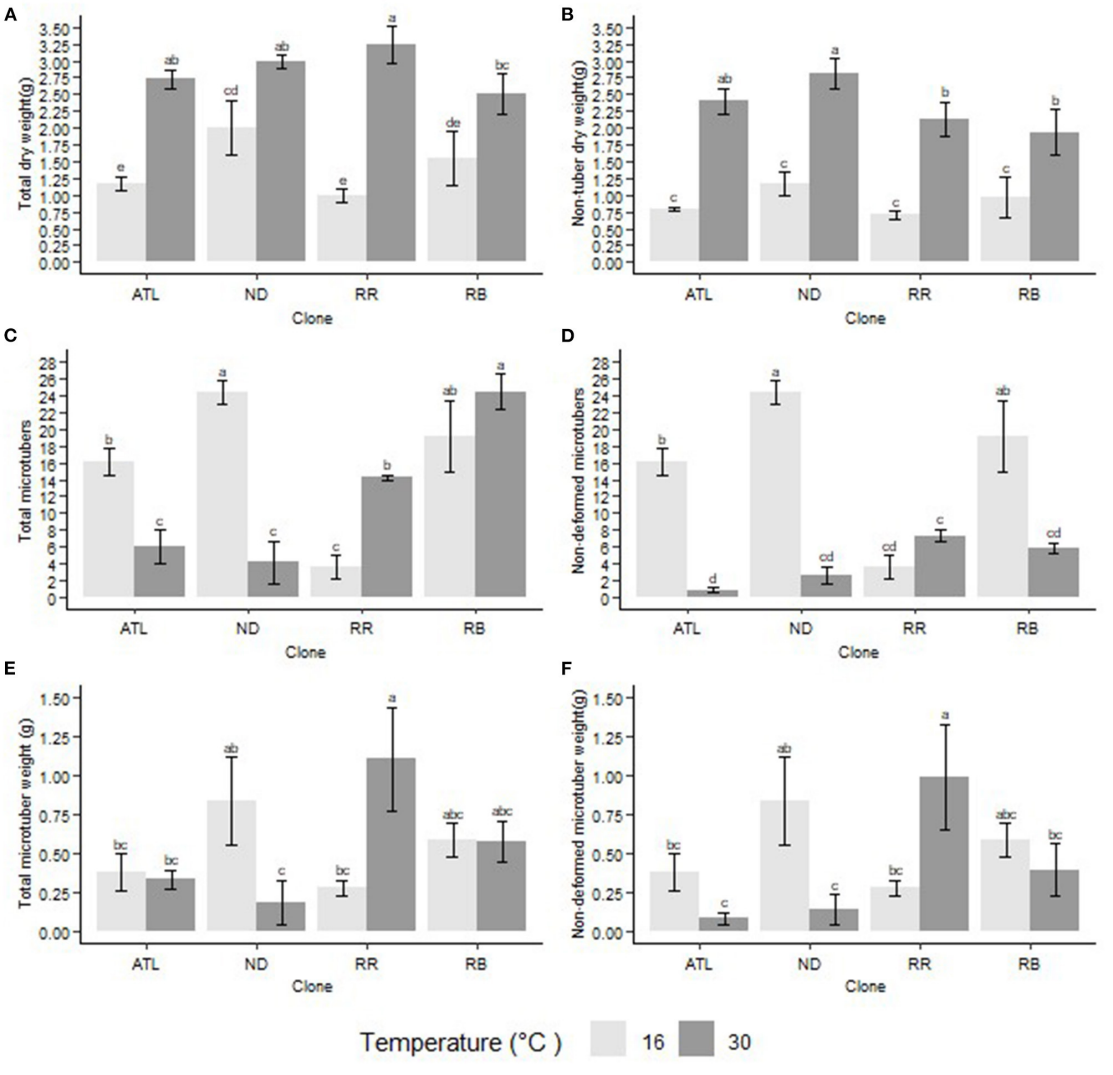
Interaction of temperature and clone on different traits. **(A)** Total biomass (dry) per explant, **(B)** Non-tuber biomass (dry) per explant, **(C)** Total microtuber number per explant, **(D)** Non-deformed microtuber number per explant, **(E)** Total microtuber weight (dry) per explant, **(F)** Non-deformed microtuber weight (dry) per explant. The following potato clones were used in the experiment: ATL (Atlantic), ND (NDTX081648CB-13W), RR (Reveille Russet), and RB (Russet Burbank). Clones with the same letter were not significantly different at *p* ≤ 0.05.

Second bioreactor trial: Similarly to our first bioreactor trial, the clones showed differential responses for total biomass production under different temperature treatments. Reveille Russet produced the highest total biomass (3.234 g per explant) at 30°C, but it showed the least total biomass (0.988 g per explant) at 16°C. Overall, there was an increase in total biomass at 30°C as compared to 16°C (Figure 1).

#### Non-tuber Biomass

First bioreactor trial: There was significant interaction between temperature and clones, indicating that clones responded differently to the temperatures tested. Also, significant differences were observed between temperatures for non-tuber biomass production. Non-tuber biomass was higher (0.668 g per explant) at 30°C than at 16°C (0.4774 g per explant) (data not shown). Potato clones were significantly different for non-tuber biomass production (both at optimum and heat-tress conditions). NDTX081648CB-13 W showed the highest non-tuber biomass production per explant (1.051 g), whereas Reveille Russet showed the lowest non-tuber biomass (0.102 g) per explant.

Second bioreactor trial: There were no significant interaction effects observed between temperature and clones, indicating that clones were responding similarly to the temperatures. But separate significant temperature and clone effects on the non-tuber biomass production were observed. Non-tuber biomass was higher (2.317 g per explant) at high (30°C) temperature, as compared with that of low (16°C) temperature (0.907 g per explant) (data not shown.). Similarly, clones also differed from each other in their non-tuber biomass production. NDTX081648CB-13 W showed the higher non-tuber biomass production per explant (1.986 g) (data are not shown) than the other clones.

#### Total Microtuber Number

First bioreactor trial: The clones responded differently to different temperatures in their capacity to produce microtubers. Significant reduction in microtuber production at 30°C was observed in all the clones (Table 3). The most significant reduction of microtuber was observed in Atlantic, followed by NDTX081648CB-13W and Russet Burbank.

**Figure 2:**
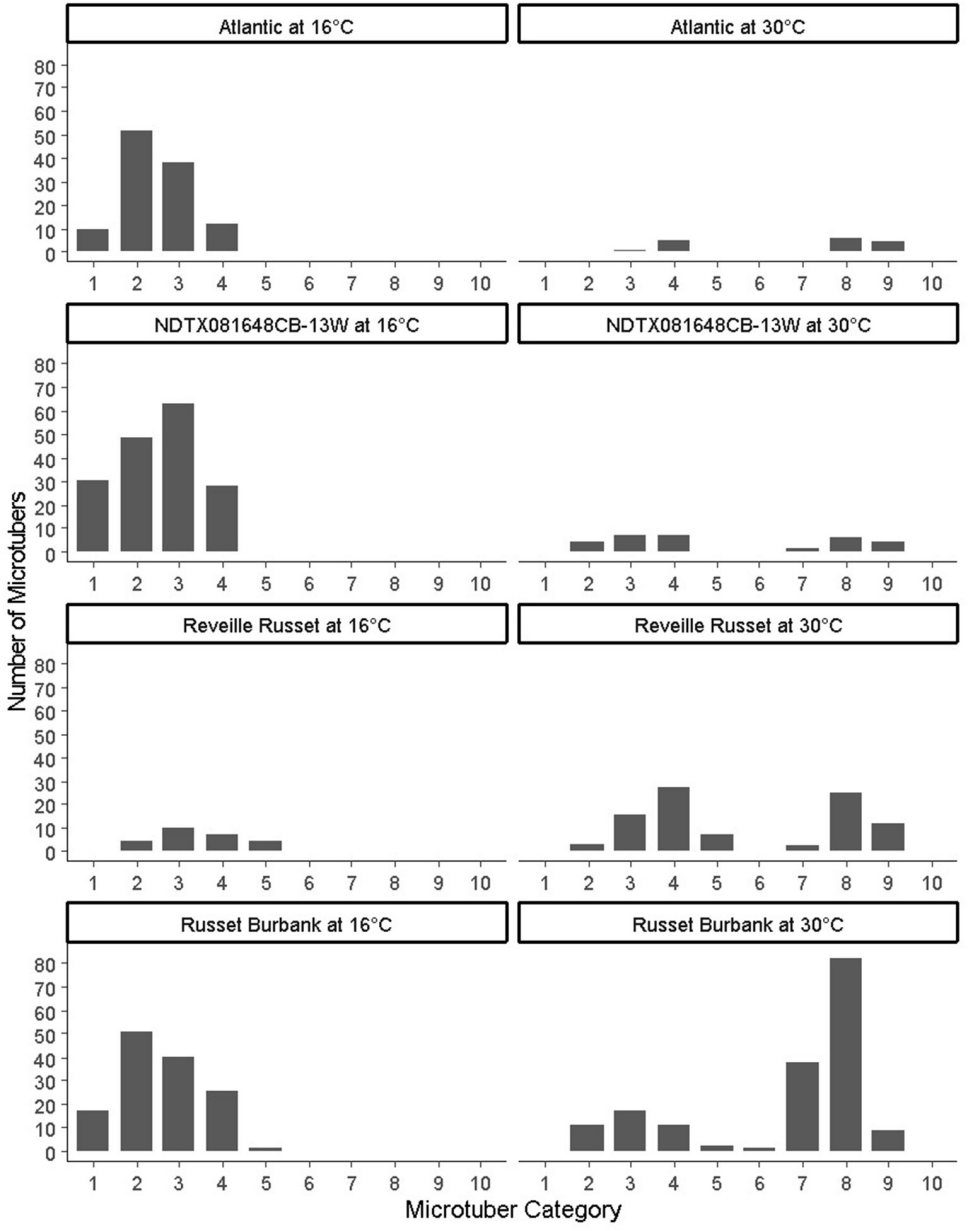
Distribution of microtubers per bioreactor based on the following categories: (1–5) indicate the size (diameter) of non-deformed microtubers (1: up to 2 mm, 2: 2–4 mm, 3: 4–8 mm, 4: 8–20 mm, 5:>20 mm), (6–10) indicate the size of deformed microtubers (6: up to 2 mm, 7: 2–4 mm, 8: 4–8 mm, 9: 8–20 mm, 10:>20 mm).

Second bioreactor trial: Clones showed differential response to different temperatures in their capacity to produce microtubers. At 30°C, the total microtuber number produced was reduced in Atlantic and NDTX081648CB-13W **(Figures 1, 2)** but increased in Russet Burbank and Reveille Russet. Microtubers produced under 16°C and 30°C were at par with Russet Burbank. However, Reveille Russet, produced a significantly higher number of microtubers at 30°C than at 16°C. The highest number of microtubers were observed in NDTX081648CB-13W at 16°C and in Russet Burbank at 30°C (Figure 1). Reveille Russet produced a similar or significantly higher number of microtubers at the higher temperature, which could be considered a sign of heat tolerance.

#### Total Microtuber Weight

First bioreactor trial: The clones responded differently to incubation temperatures in their capacity of producing microtuber biomass per explant. Significant reduction in dry weight (dry matter) production of tubers was observed in Atlantic and NDTX081648CB-13W at high temperature (Table 3). Russet Burbank had similar microtuber biomass production at both temperatures. However, Reveille Russet produced higher microtuber biomass at 30°C (0.376 g per explant) than at 16°C (0.141 g per explant). This high microtuber biomass of Reveille Russet at 30°C is similar to microtuber biomass of NDTX081648CB-13W at 16°C (Table 3).

Second bioreactor trial: Clone and temperature conditions showed significant interaction in their capacity to produce microtuber biomass. Significant reduction in dry weight (dry matter) production of tubers was observed in NDTX081648CB-13W at 30°C, but not in Atlantic and Russet Burbank (Figure 1). However, similar to the first bioreactor trial, Reveille Russet produced more dry matter, as observed by high dry weight (1.101 g per explant), compared with all other treatments and itself (0.280 g) under 16°C.

#### Non-deformed Microtuber Number and Size

**Figure 3:**
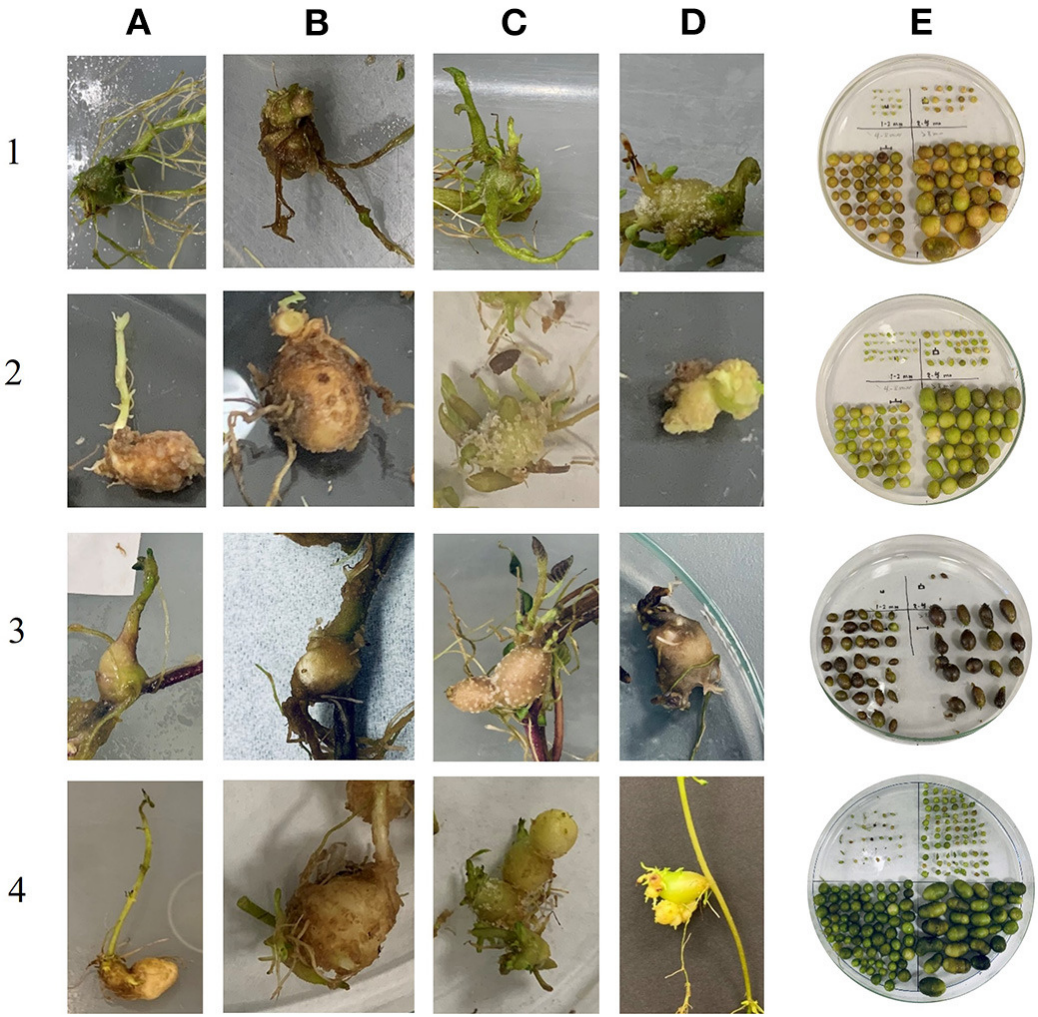
Phenotypes of deformed microtubers and non-deformed microtubers. Horizontal panels **(1–4)** represent the clones: **(1)** Atlantic, **(2)** NDTX081648CB-13W, **(3)** Reveille Russet, and **(4)** Russet Burbank. Vertical panels **(A–D)** represent the categories of deformed microtubers at 30°C and **(E)** represents non-deformed microtubers produced at 16°C. **(A)** Deformed microtuber with sprouts, **(B)** Deformed microtuber with roots, **(C)** Deformed microtuber with excessive outgrowths, **(D)** Deformed microtuber with unusual shape.

In the first bioreactor trial, the microtubers produced were not categorized as deformed or well-formed (non-deformed) but they were classified in the second one. Thus, the data for these categories are presented only for the second bioreactor trial. The reason for making this classification is that there were clear indications of tubers being deformed in higher temperatures (Figure 3). During the first bioreactor trial and literature reviews on heat stress on potatoes, observations indicated the need for a classification of the microtubers into deformed and non-deformed in bioreactors, similarly to what was observed in the field. Clones differed in their responses for the number of non-deformed tubers being produced at different temperatures. The highest number of non-deformed microtubers per explant was produced by NDTX081648CB-13W (24) at 16°C, whereas the lowest by Atlantic (1) at 30°C (Figures 1, 2). All clones, except Reveille Russet, produced a higher number of non-deformed tubers at lower optimal temperature. Reveille Russet seemed different, since it produced a statistically similar number of non-deformed microtubers in the higher temperature, as compared to the lower temperature (Figure 1).

Temperature also had an effect on the microtuber size distribution (Figure 2). In Atlantic, NDTX081648CB-13W, and Russet Burbank, at 16°C, abundant small size normal microtubers (young microtubers) were observed at the time of harvest; however, their number was reduced or absent at 30°C; in contrast, for Reveille Russet, most of the microtubers were large at both 16 and 30°C.

#### Non-deformed Microtuber Weight

All the clones, with the exception of Reveille Russet, produced higher dry weight of non-deformed microtubers at 16°C than at 30°C. NDTX081648CB-13W showed significant loss in dry weight of microtubers at 30°C. Lower dry mass of non-deformed microtubers were observed also in Atlantic and Russet Burbank though not statistically different. Contrary to the other clones, Reveille Russet produced more microtuber dry matter per explant at 30°C (8.306 g) than at 16°C (1.710 g) (Figure 1). Reveille Russet's biomass of microtubers at 30°*C* equaled or surpassed the production of biomass of any other clones at 16°C. This result may suggest that Reveille Russet can partition more assimilate toward its microtubers at higher temperatures than in lower optimum temperatures.

## Discussion

### Media Experiment

Previous studies on media selection for microtuber induction *in vitro* in potatoes indicate that higher percentage content of sucrose gave satisfactory results (Hossain et al., 2017; Pantelić et al., 2018; Guedes et al., 2019). Eight percent (8%) sucrose was found to be favorable for microtuber induction. Increasing or lowering sucrose from 8% was found to delay the microtuberization process (Hossain et al., 2017). The results obtained in the current study were similar to Gopal et al. (1998), where they found significant interaction between media and clones used. They further recommended that the media devoid of growth regulators would be desirable to look for innate capacity of genotypes to produce microtubers, thereby removing any undesirable effects of growth regulators on morphology, dormancy, or sprouting. Singh et al. (2016) also suggested that use of hormone-free microtuberizing media be used to develop a reliable *in vitro* assay for heat tolerance. Current study results show that media without hormones, in general, produced more microtubers and increased the size of microtubers. Considering the results obtained and the objective of exploring the innate capacity of clones under heat stress in bioreactors, media with 8% sucrose was selected for further experiments.

In this study, *in vitro* thermotolerance was analyzed for four potato cultivars, assessing the growth and production of microtubers of each cultivar at an elevated temperature. High temperature (30°C) was used in the study as stress treatment, compared to 16°C, which was considered the normal tuberizing temperature. Researchers have used similar or lower temperatures for heat treatment in related studies. Pantelić et al. (2018) used 29°C for their experiment on *in vitro* tuberization at high temperatures. Guedes et al. (2019) used 25°C for their experiment. Harvey et al. (1988) used 26°C. Nowak and Colborne (1989) used 28 and 30°C for their experiment. Khan et al. (2015) used 25–27°C in their experiment.

### Bioreactor Experiments

#### Total Biomass

Potatoes did not differ for total biomass grown under high temperatures in experiments conducted by Kim and Lee (2019). However, reduction in total biomass produced under high temperature has been reported (Aien et al., 2017). Total biomass reduction has been proposed to occur due to reduction in tuber biomass rather than above-ground vegetative matter in the case of potatoes grown in greenhouses or under field conditions. However, our results indicate that the total biomass has increased under high temperature. This increase was mainly due to non-tuber biomass. Singh et al. (2016) also reported that high temperature increased the total biomass in their *in vitro* microtuberization study. Similar results were obtained in both bioreactor trials, where total biomass at 30°C for all the clones was significantly higher than that of 16°C. The increase was much larger in the case of Reveille Russet, and the increase in total biomass in the case of Reveille Russet can be attributed to an increase in microtuber biomass at 30°C rather than an increase in non-tuber biomass. The total biomass increase indicates that the growth of the plant was enhanced at higher temperatures, and assimilates were used. However, the site where the accumulation occurs is a crucial point to consider.

#### Non-tuber Biomass

Non-tuber biomass significantly increased at higher temperatures. Several reports are available to validate the finding. It is thus inferred that photosynthate allocation or partitioning is critical for potatoes grown under high temperatures. Clonal variation can be expected and was found in both experiments. Under high temperature, haulm growth is accelerated, with assimilates partitioned more toward the haulm (Basu and Minhas, 1991). When grown at high temperatures (30/20°C day/night), potatoes showed that biomass allocation shifted away from tubers and toward leaves (Hancock et al., 2014). We could observe similarities in our bioreactor experiment results with that from the previous field or greenhouse studies.

#### Total Microtuber Number

There are reports of general decrease in total tuber number produced under heat stress. The number of microtubers was reduced when produced under constant 28°C as compared to continuous 20°C (Wang and Hu, 1982). High temperature (28/25°C day/night) with long photoperiod (16/8 day/night) were found to produce diminished microtuber numbers and weight than with low temperature (20/18°C day/night) with short photoperiod (14/10 day/night) (Gopal et al., 1998).

The decrease in total microtubers at higher temperatures was expected for the clones tested. One of the expectations through these experiments was that heat-tolerant clones could maintain the level of microtuber production at heat stress conditions. However, the clones did not meet this expectation in our first bioreactor trial. A different result in our second bioreactor trial- a reduction of microtuber number in two of the clones and an increase in the other two clones tested were observed. There was an increase in the microtuber numbers in Russet Burbank in the second bioreactor trial, and with many deformed tubers led us to categorize the tubers into malformed and non-deformed tubers.

There are also reports where researchers have found that the number of tubers might increase at a higher temperature, but the size and dry matter accumulation are decreased (Lafta and Lorenzen, 1995; Hancock et al., 2014). The total number of tubers produced under heat stress is genotype-dependent, and this could not be taken as the only indicator for heat tolerance. Our assumption about the NDTX081648CB-13W as a potential heat-tolerant clone was found otherwise as it could not produce microtubers at high temperature. However, total biomass and non-tuber biomass were higher than others. Total microtubers decreased with an increase in temperature from 20 to 25 and 30°C (Kim et al., 2012). Similar results were observed by Otroshy et al. (2009) in their trial with temperatures from 17 to 25°C. In the first bioreactor trial, Reveille Russet reduced its microtuber production at higher temperatures but not as drastically as other clones. The results from the second bioreactor trial show the increase of microtubers at higher temperature for Reveille Russet contrasting to other clones tested. Reveille Russet was an exception in the current study. It not only maintained its microtuberization capacity at higher temperatures, but also enhanced partitioning of photoassimilates toward the microtubers at higher temperatures.

#### Total Microtuber Weight

It is expected that tolerant potatoes would maintain their dry matter accumulation capacity under high temperatures similar to optimum conditions. Significant reduction in dry weight (dry matter) production of microtubers was observed in NDTX081648CB-13W in both bioreactor trials but not in Atlantic and Russet Burbank. This would indicate that NDTX081648CB-13 W is a susceptible clone for heat stress as there was reduction in microtuber numbers, which decreased the total microtuber weight under higher temperature in both trials. There was significant reduction in dry weight of microtubers in Atlantic in the first bioreactor trial but not in the second one. Though the number of microtubers differs significantly in the case of Atlantic, the non-significant microtuber weight of Atlantic at both temperatures would indicate that Atlantic stored its assimilates in fewer microtubers (Figure 2). Russet Burbank had similar dry matter production at both temperatures in both bioreactor trials. Reveille Russet showed a consistent increase in dry matter of microtubers in both trials at higher temperature, compared with all other treatments and itself at 16°C. These results indicate that Reveille Russet and Russet Burbank can maintain partitioning of the assimilates to the tubers even at higher temperatures. However, total microtuber weight, like total microtuber number, would not be reliable as an indicator of heat tolerance. We observed deformities, such as misshaped microtubers and excessive sprouting (equivalent to heat sprouts in the field) for Russet Burbank at 30°C. Physiological deformations of tubers, such as elongated tubers, bottlenecks, second-growth (chain tuberization), and gemmations, occur in potatoes grown in heat-stressed condition (Rykaczewska, 2015). Therefore, in the second bioreactor trial, the microtubers were classified as non-deformed and deformed tubers. It was surprising that Reveille Russet accumulated more dry matter under high temperature than under lower temperature conditions.

#### Non-deformed Microtuber Number

Rykaczewska (2015) reported that it is essential to assess and incorporate the occurrence of tuber defects into heat tolerance studies of potatoes, where total yield alone cannot be the only indicator of tolerance. Marketable tuber reductions have been reported in potatoes under higher temperatures (Aien et al., 2017; Kim et al., 2017). Non-deformed microtubers can be considered equivalent to marketable tubers from the field. Considering the total number of microtubers or total weight as indicators of heat tolerance, might lead to wrong conclusions. Russet Burbank produced microtubers in similar numbers at both conditions. However, it had a greater number of deformed tubers at higher temperature, indicating that it could not tolerate heat and maintain tuber quality. Russet Burbank has long been reported as heat susceptible (Nowak and Colborne, 1989; Gawronska et al., 1992; Ahn et al., 2003; Demirel et al., 2020). NDTX081648CB-13W and Atlantic could be easily regarded as susceptible, based on the results for non-deformed tubers at 30°C, compared to 16°C, as they produced very few microtubers at higher temperatures in contrast to lower temperatures. All the clones, except Reveille Russet, had a higher number of non-deformed tubers at the optimal temperature. Reveille Russet seemed different since it produced a statistically similar number of non-deformed microtubers in higher temperatures (7) than itself in lower temperatures (4). Microtubers were separated into deformed and non-deformed, based on visual observations, such as sprouting and misshaped microtubers with bioreactors. This distinction was not possible in a conventional *in vitro* system with liquid or solid media and gave the bioreactors an advantage to conduct heat tolerance studies.

#### Non-deformed Microtuber Weight

Considering the dry matter of microtubers produced under contrasting temperatures, Reveille Russet increased the weight of total microtubers (First bioreactor trial) and non-deformed microtubers (Second bioreactor trial) even at higher temperatures. This result suggests that Reveille Russet can partition more assimilate toward its microtubers at higher temperatures than in lower optimum temperatures. The observation of higher microtuber dry weight could be assigned to its capacity to produce numerous large size tubers (non-deformed) and less deformed ones in the case of Reveille Russet while the production of either fewer microtubers (Atlantic and NDTX081648CB-13W) or more deformed tubers (Russet Burbank) in case of other clones at higher temperatures. Reveille Russet's results were similar to those obtained using the potato clone AVRDC-1287.19, which produced more dry weight in the hot chamber than the cool chamber (Midmore and Prange, 1991). Since the ultimate goal of heat tolerance in potatoes would be producing high-quality potatoes without deformities, traits like the non-deformed microtuber number and microtuber weight can be reliable criteria to judge a clone's heat tolerance capacity in similar experiments. The results for the non-deformed tubers were still consistent with Reveille Russet, as it had a higher number of non-deformed microtubers that weighed more than any of the other treatments.

## Conclusions

Media with 8% sucrose can induce microtubers in solid as well as liquid medium. Producing numerous tubers *in vitro* with high accumulation of dry matter is not enough to declare a potato clone as heat tolerant. In fact, if the tubers are deformed, the clones will be reported as heat susceptible. The remarkable capacity of Reveille Russet to produce a high number of non-deformed microtubers weighing more than any of the other clones independently of the temperature treatment indicates that it possesses very strong thermotolerance. Thus, Reveille Russet can be used as a thermotolerant check for heat stress screening of potato clones. Follow-up studies should focus on understanding the underlying genetic, morphological, or physiological reasons for the extraordinary thermotolerance observed in Reveille Russet.

Bioreactors seem to be an efficient system to screen stress tolerance for plants that can be grown *in vitro*. The system allows switching from growth media to tuberization media without disrupting plants. It also enables the quantification and categorization of tubers into deformed and non-deformed. The distinction between well-formed vs. mishappen tubers is possible under field conditions but very challenging under conventional *in vitro* systems using solid or liquid media in small tubes or flasks. This possibility of categorizing microtubers into different classes will let us exploit traits like non-deformed microtuber number and non-deformed microtuber weight as reliable indicators for heat tolerance in potatoes under a similar setup. Bioreactors can be used to screen selected clones and confirm their thermotolerance *in vitro*. The identification of thermotolerant potato clones can lead to building a successful breeding program for heat tolerance and yield an increase in potato in the face of global warming.

## Data Availability Statement

The raw data supporting the conclusions of this article will be made available by the authors, without undue reservation.

## Author Contributions

MV, JS, and KM conceived and designed experiments, supervised experiments, and guided data analysis. SG, NS-G, and MT performed experiments and analyzed data. All authors wrote the manuscript, read the final version, and approved submission for publication.

## Conflict of Interest

The authors declare that the research was conducted in the absence of any commercial or financial relationships that could be construed as a potential conflict of interest.

## Publisher's Note

All claims expressed in this article are solely those of the authors and do not necessarily represent those of their affiliated organizations, or those of the publisher, the editors and the reviewers. Any product that may be evaluated in this article, or claim that may be made by its manufacturer, is not guaranteed or endorsed by the publisher.
